# 

**Published:** 1896-01

**Authors:** 


### CONTENTS.

ORIGINAL ARTICLES—

| | |
|---|---|
| Ligation of the Left Subclavian Arteries (Third Division) and the Transversalis Colli and Suprascalpular, with Cocaine Anæsthesia. Subsequent Bloodless Amputation at the Shoulder Joint. By John A. Wyeth, New York.......... .. | 1 |
| Operation for Appendicitis. By W. O. Roberts, M. D., Louisville, Ky ...... ...... ........................ .................. | 5 |
| Extra-Peritoneal Lipoma. By W. H. Wathen, A. M., M. D., Louisville, Ky........................................ ..... | 7 |

Malarial Origin of Rheumatism. By R. L. Patterson, M. D., Coyville, Kas .......... 8

SOCIETY REPORTS—

Southern Surgical and Gynæcological Association .......... 9

EDITORIAL.

To Subscribers .......... 42

Special Notes .......... 44

Prescriptions .......... 50

